# Flexizyme-catalyzed synthesis of 3′-aminoacyl-NH-tRNAs

**DOI:** 10.1093/nar/gkz143

**Published:** 2019-03-07

**Authors:** Takayuki Katoh, Hiroaki Suga

**Affiliations:** Department of Chemistry, Graduate School of Science, The University of Tokyo, 7-3-1 Hongo, Bunkyo-ku, Tokyo 113-0033, Japan

## Abstract

Structural analysis of ribosomes in complex with aminoacyl- and/or peptidyl-transfer RNA (tRNA) often suffers from rapid hydrolysis of the ester bond of aminoacyl-tRNAs. To avoid this issue, several methods to introduce an unhydrolyzable amide bond instead of the canonical ester bond have been developed to date. However, the existing methodologies require rather complex steps of synthesis and are often inapplicable to a variety of amino acids including those with noncanonical structures. Here, we report a new method to synthesize 3′-aminoacyl-NH-tRNAs by means of flexizymes—ribozymes capable of charging amino acids onto tRNAs. We show that two types of flexizymes, dFx and eFx, are able to charge various amino acids, including nonproteinogenic ones, onto tRNA or microhelix RNA bearing the 3′-deoxy-3′-amino-adenosine. Due to the versatility of the flexizymes toward any pair of nonproteinogenic amino acids and full-length or fragment tRNAs, this method provides researchers an opportunity to use a wide array of hydrolytically stable 3′-aminoacyl-NH-tRNAs and analogs for various studies.

## INTRODUCTION

Aminoacyl-tRNA is a key molecule for messenger RNA decoding and translation of codons into the corresponding amino acids. The amino acid moiety is linked to the 3′-end adenosine of the cognate transfer RNAs (tRNAs) via an ester bond, which is catalyzed by aminoacyl-tRNA synthetases (ARSs) in cells. In ribosomal translation, peptide chains are elongated by repeating peptidyl transfer from the P-site peptidyl-tRNA to the A-site aminoacyl-tRNA, translocation of those tRNAs, and accommodation of the next aminoacyl-tRNA onto the A site. Therefore, in order to understand the mechanism of ribosomal translation, structural analysis of ribosome complexed with aminoacyl- and/or peptidyl-tRNA is requisite. X-ray crystallography and cryo-electron microscopic analysis are often used for such purposes. However, the insufficient hydrolytic stability of the ester bond of aminoacyl-tRNA cannot assure the analysis of ribosome in complex with aminoacyl-tRNA and/or peptidyl-tRNA (1). Such an issue could be overcome by an appropriate introduction of the amide bond instead of the canonical ester bond in aminoacyl-tRNA (2,3). Here we refer to the molecule, where an amino acid was charged onto 3′-deoxy-3′-amino-tRNA, as 3′-aminoacyl-NH-tRNA for simplicity, whereas the canonical aminoacyl-tRNA is referred to as 3′-aminoacyl-O-tRNA. Similarly, 3′-deoxy-3′-amino-tRNA and the canonical tRNA are referred to as 3′-NH_2_-tRNA and 3′-OH-tRNA, respectively. To date, because of technical difficulty in the preparation of a full-length 3′-aminoacyl-NH-tRNA, a chemically accessible 3′-aminoacyl-NH-tRNA analog, e.g. only the 3′-end short fragment of 3′-aminoacyl-NH-tRNA (3′-aminoacyl-NH-sfRNA), such as CC-puromycin, was used for structural and functional analysis of ribosome complexes (4–8).

Several methods have been devised to prepare 3′-aminoacyl-NH-tRNA or its analogs. A classic method reported in the 1970s utilized ARSs for the preparation of 3′-aminoacyl-NH-tRNAs (9,10). Since the charging efficiency of cognate amino acids onto tRNAs was unfortunately rather low (e.g. 22 and 8% for charging Arg and Pro, respectively) (11), this method has not been generally used for the preparation of 3′-aminoacyl-NH-tRNAs in conjunction with studies on the ribosome. Moreover, the substrate specificity of natural ARSs limits applications to the use of cognate tRNAs and amino acids. Chemical synthesis approaches were also developed to prepare 3′-aminoacyl-NH-sfRNAs (12–21). Strazewski *et al.* synthesized 3′-*O*-methyltyrosyl-NH-sfRNAs, puromycin analogs, by phosphoramidite-based oligoribonucleotide synthesis on a derivative of 3′-deoxy-3′-*O*-methyltyrosylamino-adenosine immobilized on a solid support (12,13). They also utilized a 3′-deoxy-3′-alanylamino-adenosine derivative as the first building block, and performed FMOC-based oligopeptide synthesis followed by oligoribonucleotide synthesis to prepare 3′-peptidyl-alanyl-NH-sfRNAs (14). Later on, Micura’s group expanded the scope of available amino acids by using a 3′-deoxy-3′-azido-adenosine derivative as the intermediate, which enables coupling of diverse amino acids on the azide residue at a later step of the synthesis (15–17). However, the above methods involved rather laborious chemical synthetic steps and are not readily accessible to the derivatization of full-length tRNAs (18).

Flexizymes are versatile ribozymes capable of synthesizing aminoacyl-tRNA using pre-activated amino acid substrates (22–28). Compared with ARSs, aminoacylation occurs specifically at the 3′-position of the 3′-terminal adenosine, while class I enzymes aminoacylate at the 2′ and class II at the 3′-position (23). Flexizymes were evolved from pools of random RNA sequences through *in vitro* selection, and currently there are several variants of flexizymes, including eFx and dFx. Substrates of eFx are amino acid cyanomethyl esters (CMEs) or 4-chlorobenzyl thioesters (CBTs), while dFx utilizes amino acid dinitrobenzyl esters (DBEs). Since eFx and dFx recognize only the conserved 3′-terminal CCA region of tRNAs, any type of tRNA or shorter RNAs with CCA ends can be used as substrates. Moreover, eFx and dFx are able to charge nonproteinogenic aminoacyl-donors onto tRNAs. In fact, using such nonproteinogenic aminoacyl-tRNAs, we have demonstrated ribosomal synthesis of peptides containing a wide array of nonproteinogenic amino acids beyond proteinogenic ones (25,29–37). Although flexizymes were originally developed for charging amino acids onto 3′-OH-tRNA and therefore have not been applied to aminoacylation of 3′-NH_2_-tRNAs nor 3′-NH_2_-sfRNAs, flexizymes are assumed to have this potential.

Here, we report an application of the flexizyme method to the synthesis of full-length 3′-aminoacyl-NH-tRNAs or microhelix RNAs. As it overcomes limitations in the choice of amino acids and tRNAs, this concept gives us new opportunities to pursue structural analyses of 3′-aminoacyl-NH-tRNAs when bound to the ribosome, which includes the use of nonproteinogenic aminoacyl-tRNA analogs such as d-aminoacyl-NH-tRNAs and *N*-methyl-aminoacyl-NH-tRNAs.

## MATERIALS AND METHODS

### Preparation of flexizymes

Flexizymes (dFx and eFx) were prepared by *in vitro* transcription using T7 RNA polymerase. Template DNAs coding the flexizymes were prepared by extension of forward and reverse extension primer pairs, and polymerase chain reaction (PCR) using forward and reverse PCR primer pairs (See Supplementary Table S1 for the sequences of primers). About 5 cycles or 12 cycles of 40 s at 94°C, 40 s at 50°C and 40 s at 72°C were carried out for the extension reaction and PCR, respectively. The resulting PCR products were purified by phenol/chloroform extraction and ethanol precipitation, and then resuspended in water. The template DNAs encode a T7 promoter at the 5′ end and the downstream flexizyme sequence (See Supplementary Table S1 for the sequences). Transcription was carried out at 37°C for 16 h in a 500 μl reaction mixture (40 mM Tris–HCl (pH 8.0), 22.5 mM MgCl_2_, 10 mM dithiothreitol (DTT), 1 mM spermidine, 0.01% Triton X-100, 0.12 μM T7 RNA polymerase, 0.04 U/μl RNasin RNase inhibitor (Promega) and 5 mM NTP mix). Then, the RNA transcripts were subjected to RQ1 DNase (Promega) treatment for 30 min at 37°C, and purified on 12% polyacrylamide gels containing 6 M urea.

### Preparation of 3′-NH_2_-tRNA

*Escherichia coli* tRNA^Tyr^ that lacks the 3′-end adenosine was synthesized by *in vitro* transcription using a protocol similar to that used for the flexizyme preparation. First, template DNAs were prepared by overlap extension and PCR using specific primer sets (See Supplementary Table S1 for the sequences of primers). About 5 cycles or 14 cycles of 40 s at 94°C, 40 s at 50°C and 40 s at 72°C were carried out for the extension reaction and PCR, respectively. Note that the reverse primer for the PCR, whose sequence is 5′-GG_m_TGGTGGGGGAAGGATTC-3′, contains 2′-*O*-methylguanosine at the second nucleotide (underlined), which is required to prevent the addition of extra nucleotides at the 3′-end of the transcript caused by T7 RNA polymerase (38). T7 transcription was carried out using the same reaction mixture as for the flexizyme preparation, except that 5 mM guanosine monophosphate (GMP) was added to the above solution in order to initiate the transcription of tRNA with GMP. Then, the 3′-end adenosine bearing a 3′-amino group was added to the above transcript by tRNA nucleotidyltransferase using 3′-NH_2_-adenosine triphosphate (ATP) as substrate. Note that attachment of 3′-NH_2_-ATP to tRNA using tRNA nucleotidyltransferase has been previously demonstrated elsewhere (1,9). For preparation of the recombinant tRNA nucleotidyltransferase, *Archaeoglobus fulgidus* tRNA nucleotidyltransferase gene (39) was cloned into the pET-22b vector, which encodes a hexa-histidine tag, and introduced into *E. coli* BL21(DE3)pLysS for overexpression, followed by histidine tag purification using a HisTrap HP column (GE healthcare). 3′-NH_2_-ATP was purchased from BioLog, Inc. The reaction was carried out at 37°C for 16 h in a 500-μl reaction mixture containing 2 μM tRNA, 3 mM 3′-NH_2_-ATP, 2 μM tRNA nucleotidyltransferase, 100 mM glycine-NaOH (pH 9.0), 10 mM MgCl_2_ and 1 mM DTT. Then, the reaction mixture was subjected to drop dialysis against water using a nitrocellurose membrane (Millipore, VSWP02500) in order to remove unreacted 3′-NH_2_-ATP, followed by phenol/chloroform extraction and ethanol precipitation.

### Aminoacylation by means of flexizymes

l-Phe, d-Phe, l-Tyr, l-IodoPhe, l-MePhe and l-AcPhe were activated as CMEs according to the previously reported method (22,25). Likewise, l-TfaLys, l-Thr and l-MeVal were activated as CBTs. d-Ala, l-Ser and l-Thr were activated as DBEs. For cyanomethyl esterification, 0.25 mmol α-*N*-Boc-amino acid was dissolved in 100 μl *N*,*N*-dimethylformamide (DMF), mixed with 100 μl chloroacetonitrile and 0.275 mmol triethylamine (TEA) and stirred at room temperature for 12 h. For dinitrobenzyl esterification, a mixture of 0.25 mmol α-*N*-Boc-amino acid, 0.275 mmol 3,5-dinitrobenzyl chloride and 0.275 mmol TEA in 100 μl DMF was stirred at room temperature for 12 h. For 4-chlorobenzyl esterification, 0.25 mmol α-*N*-Boc-amino acid was dissolved in 100 μl 1,4-dioxane, mixed with 0.25 mmol 4-chlorobenzylmercaptan, 0.25 mmol *N*,*N′*-dicyclohexylcarbodiimide in 200 μl ethylacetate and stirred at room temperature for 3 h. Then, 4 ml diethyl ether was added to the reaction mixture and the solution was washed with 1 M HCl (3 ml ×2), saturated NaHCO_3_ in water (3 ml ×2) and saturated NaCl in water (5 ml ×1), and the organic layer was dried over MgSO_4_ and concentrated in vacuo. For deprotection of Boc, the activated α-*N*-Boc-amino acids were dissolved in 2 ml of 4 M HCl/ethyl acetate and incubated for 20 min at room temperature. The reaction mixture was concentrated under reduced pressure, and then the product was precipitated by the addition of 3 ml diethyl ether. After removing the supernatant, the pellet was washed with additional 3 ml diethyl ether.

These activated amino acids were charged onto tRNA or a microhelix using appropriate flexizymes (eFx for CME or CBT activated ones or dFx for DBE activated ones). The 3′-amino-microhelix RNA was purchased from Gene Design, Inc. The flexizyme reactions were carried out at 4°C for 0 to 96 h in 50 mM 4-(2-hydroxyethyl)-1-piperazineethanesulfonic acid (HEPES)-KOH (pH 7.5), 600 mM MgCl_2_, 20% DMSO, 25 μM dFx or eFx, 25 μM tRNA or microhelix and 5 mM activated amino acid. For the reaction at pH 8.5, 50 mM Bicine-KOH (pH 8.5) was used instead of 50 mM HEPES-KOH (pH 7.5). Then, the resulting aminoacyl-tRNA or aminoacyl-microhelix were subjected to ethanol precipitation to remove activated amino acids, and then the pellets were washed with 70% ethanol containing 0.1 M sodium acetate (pH 5.2) twice.

### RNase T1 digestion of 3′-aminoacyl-NH-tRNAs and MALDI-TOF mass spectrometry

Digestion of aminoacyl-tRNAs by RNase T1 was carried out at 37°C for 15 min in a 25-μl solution containing 50 mM Tris–HCl (pH 7.5), 10 mM ethylenediaminetetraacetic acid, 2.5 μM aminoacyl-tRNA, 10 U RNase T1 (Thermo Fisher Scientific). Then, the aminoacyl-tRNAs were desalted with SPE C-tip (Nikkyo Technos) and co-crystalized with 3-hydroxypicolinic acid. MALDI-TOF MS analyses were carried out using UltrafleXtreme (Bruker Daltonics) by linear/positive mode. For external mass calibration, two synthetic DNAs, 5′-AGCTTGACTGCGAGCGTG-3′ ([M+H]^+^ = 5556.6) and 5′-TTAGTGCAATGGCATAAGCC-3′ ([M+H]^+^ = 6142.0), were used. MALDI-TOF MS analyses of 3′-aminoacyl-NH-microhelices were done without RNase T1 digestion in a similar way. For external mass calibration, three synthetic DNAs, 5′-ATGCCACGTACGCAGTCACGGC-3′ ([M+H]^+^ = 6706.4), 5′-ATGCCACGTACGCAGTCACGGCA-3′ ([M+H]^+^ = 7019.6), and 5′-ATGCCACGTACGCAGTCACGGCATT-3′ ([M+H]^+^ = 7628.0), were used.

### Analysis of 3′-aminoacyl-tRNA and microhelices by acid polyacrylamide gel electrophoresis

About 20 pmol 3′-aminoacyl-tRNAs or 3′-aminoacyl-NH-microhelices were analyzed on 10 or 20% polyacrylamide gels containing 50 mM sodium acetate (pH 5.2) and 6 M urea. Electrophoresis was carried out at 120 V for 45 min (tRNA) or 2.5 h (microhelix), followed by SYBR green II (tRNA) or ethidium bromide staining (microhelix) and detection using a Typhoon FLA 7000 (GE Healthcare).

### Electrophoresis mobility shift assay of EF-Tu bound to 3′-aminoacyl-NH-tRNAs

l-Ser, l-Phe and l-Tyr were charged onto 3′-NH_2_- or 3′-OH-tRNA^Tyr^ in a 96-h or a 2-h flexizyme reaction, using l-Ser-DBE, l-Phe-CME and l-Tyr-CME, respectively. A total of 10 μM EF-Tu was pre-incubated at 37°C for 10 min in a 10-μl solution containing 1 mM guanosine triphosphate (GTP), 70 mM HEPES-KOH (pH 7.6), 52 mM NH_4_OAc, 8 mM Mg(OAc)_2_, 30 mM KCl, 1 mM DTT, 6% glycerol, 10 mM phosphoenolpyruvate and 0.08 U/μl pyruvate kinase. Then, 50 pmol aminoacyl-tRNAs were mixed with 50 pmol EF-Tu in a solution containing 30 mM HEPES-KOH (pH 7.6), 39 mM NH_4_OAc and 6 mM Mg(OAc)_2_, and incubated at 37°C for 10 min for the EF-Tu/GTP/aminoacyl-tRNA ternary complex formation. The mixture was then applied to 8% native polyacrylamide gel electrophoresis (PAGE) at 4°C for 2 h at 50 V. Bands of the EF-Tu were detected by SYPRO Red staining using a Typhoon FLA 7000 (GE Healthcare).

## RESULTS

### Aminoacylation of 3′-NH_2_-tRNA by means of flexizymes

**Figure 1.**
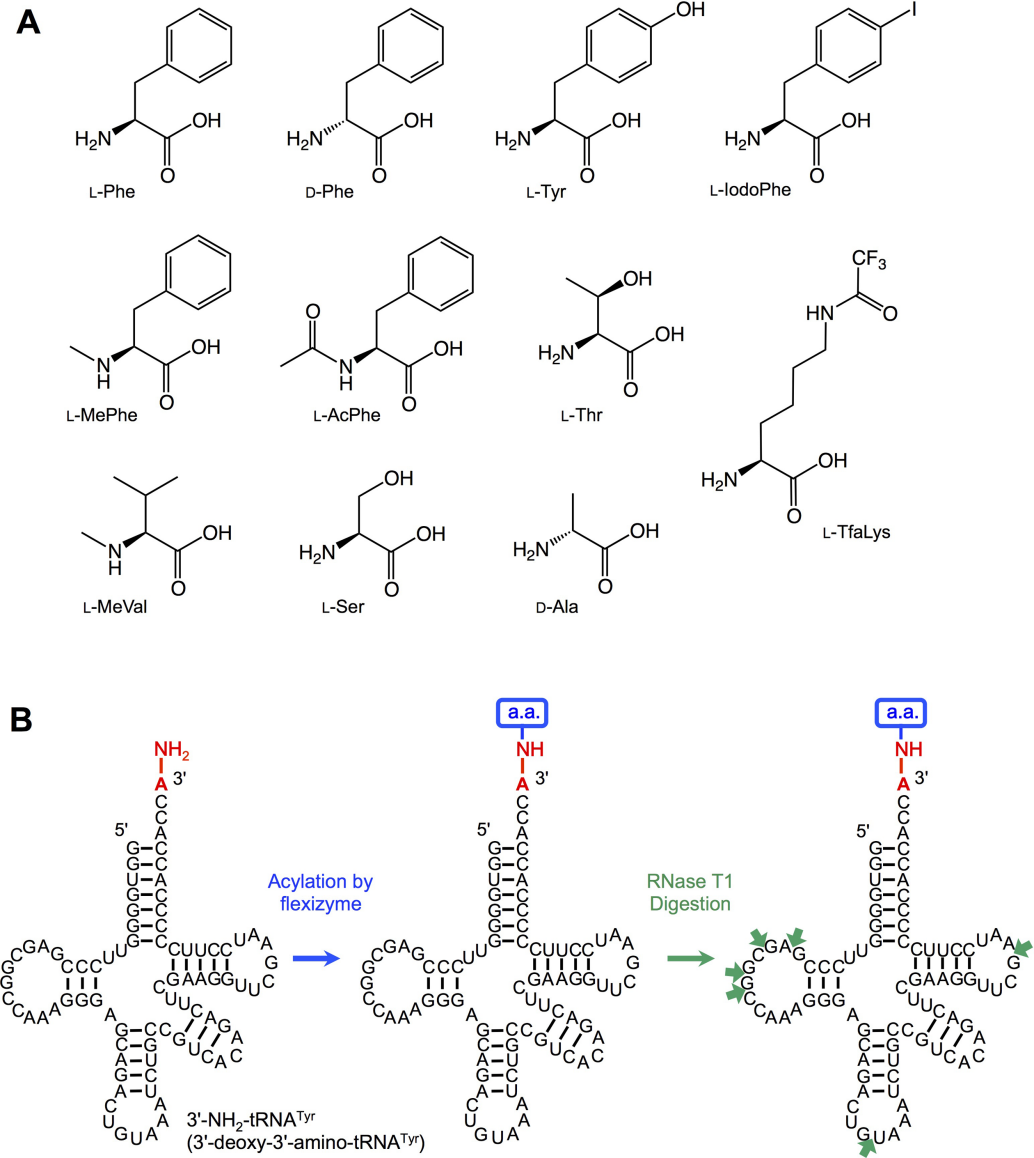
Aminoacylation of 3′-NH_2_-tRNA catalyzed by flexizymes. (**A**) Structures of amino acids used for aminoacylation in this study. (**B**) Schematic depiction of flexizyme-catalyzed aminoacylation and analysis of the resulting aminoacyl-tRNA. *In vitro* transcribed *E. coli* tRNA^Tyr^ bearing a 3′-amino group at the 3′-terminal adenosine was subjected to the flexizyme reaction using various activated amino acids. Then, the 3′-aminoacyl-NH-tRNA was digested with RNase T1 and analyzed by MALDI-TOF MS. Small green arrows indicate the cleavage sites of RNase T1, a ribonuclease specific for single-stranded G residues.

**Figure 2.**
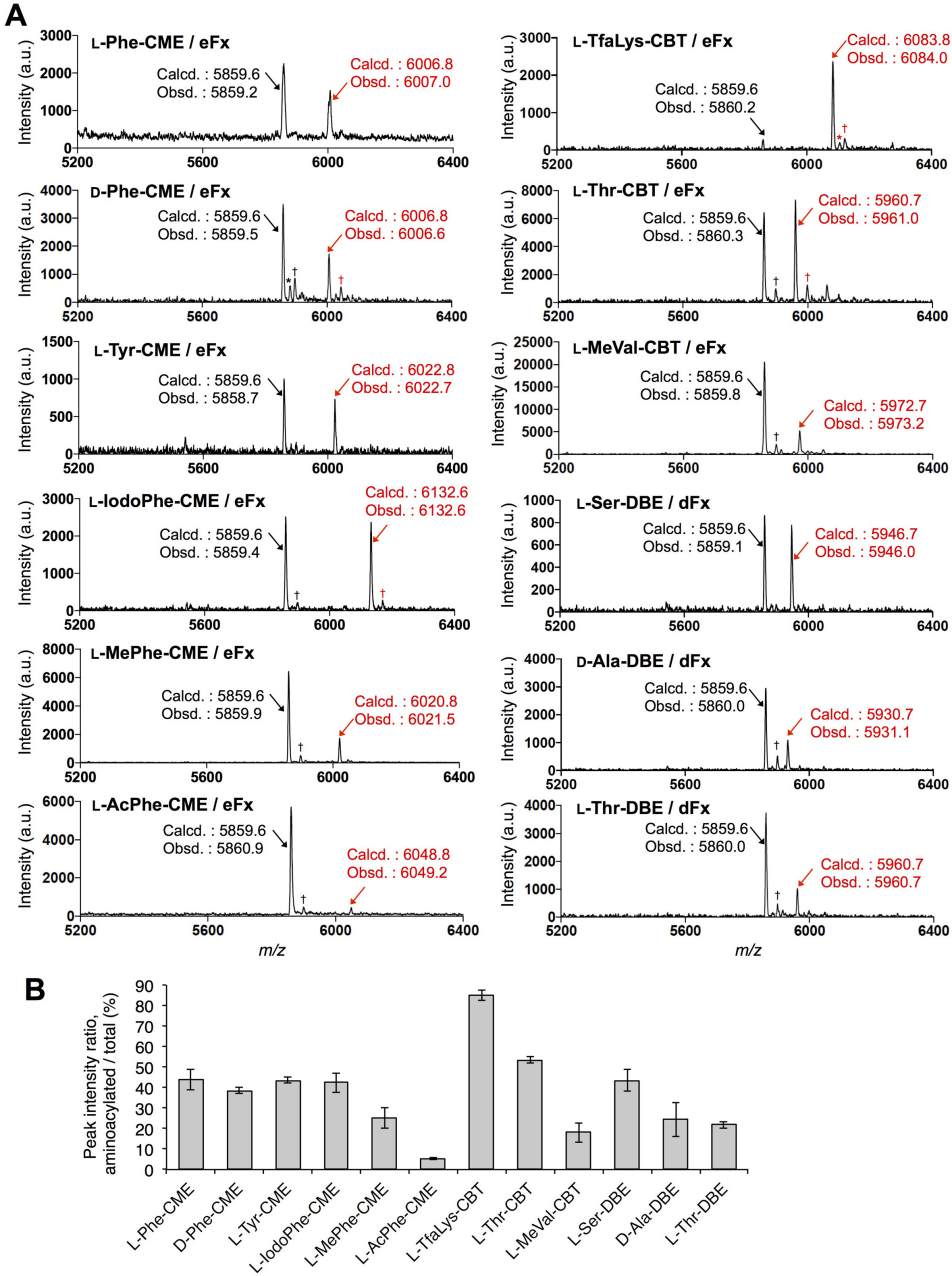
MALDI-TOF mass spectrometric analysis of aminoacylation of 3′-NH_2_-tRNA catalyzed by flexizymes. (**A**) MALDI-TOF mass spectra of RNase T1-treated 3′-aminoacyl-NH-tRNA fragments, either charged by eFx or dFx. Red arrows indicate the peaks of the fragments derived from aminoacyl-tRNA, and black arrows are those of non-acylated ones. Calculated (Calcd.) and observed (Obsd.) *m/z* values of the [M+H]^+^ ions are also shown. * and † indicate the [M+Na]^+^ and [M+K]^+^ ions, respectively. (**B**) Ratios of the peak intensities of MALDI-TOF mass spectra shown in (A). Values are calculated by the intensity of the acylated peak divided by the sum of non-acylated and acylated peak; *n* = 3, error bars; s.d.

Using two kinds of flexizymes, termed dFx and eFx, we demonstrated aminoacylation of 3′-NH_2_-tRNA with l-phenylalanine (l-Phe), d-phenylalanine (d-Phe), l-tyrosine (l-Tyr), l-*p*-iodophenylalanine (l-IodoPhe), *N*-methyl-l-phenylalanine (l-MePhe), *N*-acetyl-l-phenylalanine (l-AcPhe), *N*_ϵ_-trifluoroacetyl-l-lysine (l-TfaLys), l-threonine (l-Thr), *N-*methyl-l-valine (l-MeVal), l-serine (l-Ser) and d-alanine (d-Ala) (Figure 1A). These amino acids were pre-activated as CME forms for l-Phe, d-Phe, l-Tyr, l-IodoPhe, l-MePhe and l-AcPhe, CBTs for l-TfaLys and l-MeVal, and DBEs for l-Ser and d-Ala. For l-Thr, CBT and DBE forms were prepared. *Escherichia coli* 3′-NH_2_-tRNA^Tyr^ bearing a 3′-amino group instead of the canonical hydroxy group at the 3′-terminal adenosine (A) was prepared by an enzymatic reaction using a tRNA nucleotidyltransferase and 3′-NH_2_-ATP. The tRNA lacking the 3′-terminal A was synthesized by *in vitro* transcription, followed by the tRNA nucleotidyltransferase reaction (See ‘Materials and Methods’ section). Then, the 3′-NH_2_-tRNA was subjected to the aminoacylation using dFx or eFx and the pre-activated amino acids (Figure 1B, see ‘Materials and Methods’ section for the experimental conditions), followed by RNase T1 treatment to digest the 3′-aminoacyl-NH-tRNA at single-stranded G residues (Figure 1B, indicated by green arrows), and MALDI-TOF mass spectrometric analysis. RNase T1 treatment aimed at reducing the size of the analyte from ∼27 000 Da (full-length aminoacyl-tRNA) to ∼6000 Da (3′-terminal fragment), since the sensitivity and resolution of MALDI-TOF MS significantly drops at higher *m/z* ratios. Figure 2A shows the MALDI-TOF MS of the 3′-terminal fragments of the 3′-aminoacyl-NH-tRNAs synthesized by eFx and dFx, respectively. All the amino acid substrates were successfully charged onto the 3′-NH_2_-tRNA, although the efficiency of the aminoacylation significantly differs depending on the type of amino acids. The ratios of the peak intensities, ‘acylated’ divided by ‘non-acylated + acylated’, are summarized in figure 2B. l-TfaLys showed the highest ratio (85%), whereas l-AcPhe showed the lowest ratio (5%). The average ratio of the 12 substrates was 37%. Comparing the aminoacylation levels of the six Phe analogs, l-Phe, d-Phe, l-Tyr and l-IodoPhe showed similar values (44, 39, 44 and 42%, respectively), whereas l-MePhe and l-AcPhe showed significantly lower values (25 and 5%, respectively), indicating that the side-chain modifications and the difference of l/d-configuration have less effect on the acylation efficiency than the amino group modifications.

**Figure 3.**
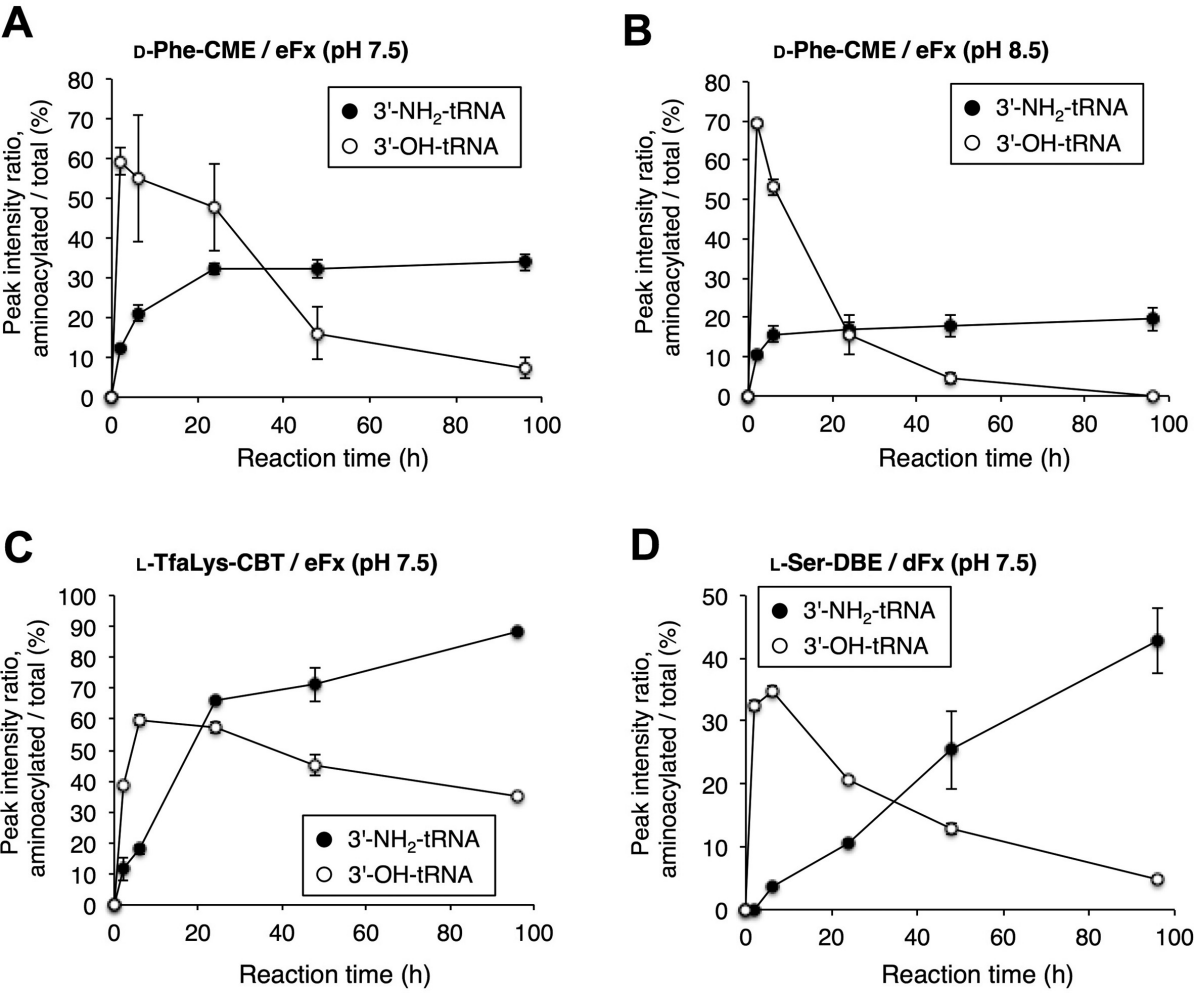
Time-course analysis of aminoacylation catalyzed by flexizymes. (**A**) d-Phe-CME at pH 7.5. (**B**) d-Phe-CME at pH 8.5. (**C**) l-TfaLys-CBT at pH 7.5. (**D**) l-Ser-DBE at pH 7.5. d-Phe-CME and l-TfaLys-CBT were charged by eFx, and l-Ser-DBE by dFx onto *E. coli* tRNA^Tyr^ bearing an 3′-deoxy-3′-amino group (3′-NH_2_-tRNA) or a 3′-hydroxy group (3′-OH-tRNA) at the 3′-terminal adenosine. Reactions were performed at pH 7.5 and also at pH 8.5 for d-Phe-CME. Ratios of the peak intensities of MALDI-TOF MS shown in Supplementary Figure S3 are plotted. Values are calculated by the intensity of the acylated peak divided by the sum of non-acylated and acylated peaks; *n* = 3, error bars; s.d.

We also conducted a time-course experiment using d-Phe-CME, l-TfaLys-CBT and l-Ser-DBE (Figure 3 and Supplementary Figure S1). d-Phe-CME and l-TfaLys-CBT were charged by eFx, and l-Ser-DBE by dFx under the same reaction conditions as the above experiment except for the reaction time (0, 2, 6, 24, 48 and 96 h). For d-Phe, higher pH (pH 8.5) was also tested in addition to pH 7.5. As a control, *E. coli* 3′-OH-tRNA^Tyr^ bearing a canonical hydroxy group at the 3′-terminal A was used as aminoacylation substrate. In the case of the 3′-OH-tRNA, the ratio of the desired product in the MALDI-TOF MS peaked at 2 h (d-Phe) or 6 h (l-TfaLys and l-Ser) and gradually decreased as the reaction time increased. This is presumably because a competition between aminoacylation and hydrolytic deacylation occurs. Due to the decomposition of the activated amino acids in the reaction mixture, the rate of aminoacylation gradually became slower than that of deacylation after the peak time (2 or 6 h). On the other hand, in the case of the aminoacylation using the 3′-NH_2_-tRNA, no decrease of the peak ratio was observed for any amino acid substrate even at 96 h, indicating that no deacylation occurred. For d-Phe, the ratio plateaued at 24 h (pH 7.5) or 6 h (pH 8.5), and the efficiency was better at pH 7.5 (35%) than pH 8.5 (20%). For l-TfaLys and l-Ser, the ratios were still increasing at 96 h, indicating the higher stability of the activated amino acids allowed aminoacylation to proceed. To analyze the hydrolytic stability of tRNA body under the flexizyme reaction conditions, d-Phe-O-tRNA and d-Phe-NH-tRNA were prepared by a 2-h or a 96-h reaction, respectively, at pH 7.5 using eFx, and subjected to 10% acid PAGE, showing that no significant degradation of the tRNA body nor eFx occurred (Supplementary Figure S2).

### Aminoacylation of 3′-NH_2_-microhelix RNA

**Figure 4.**
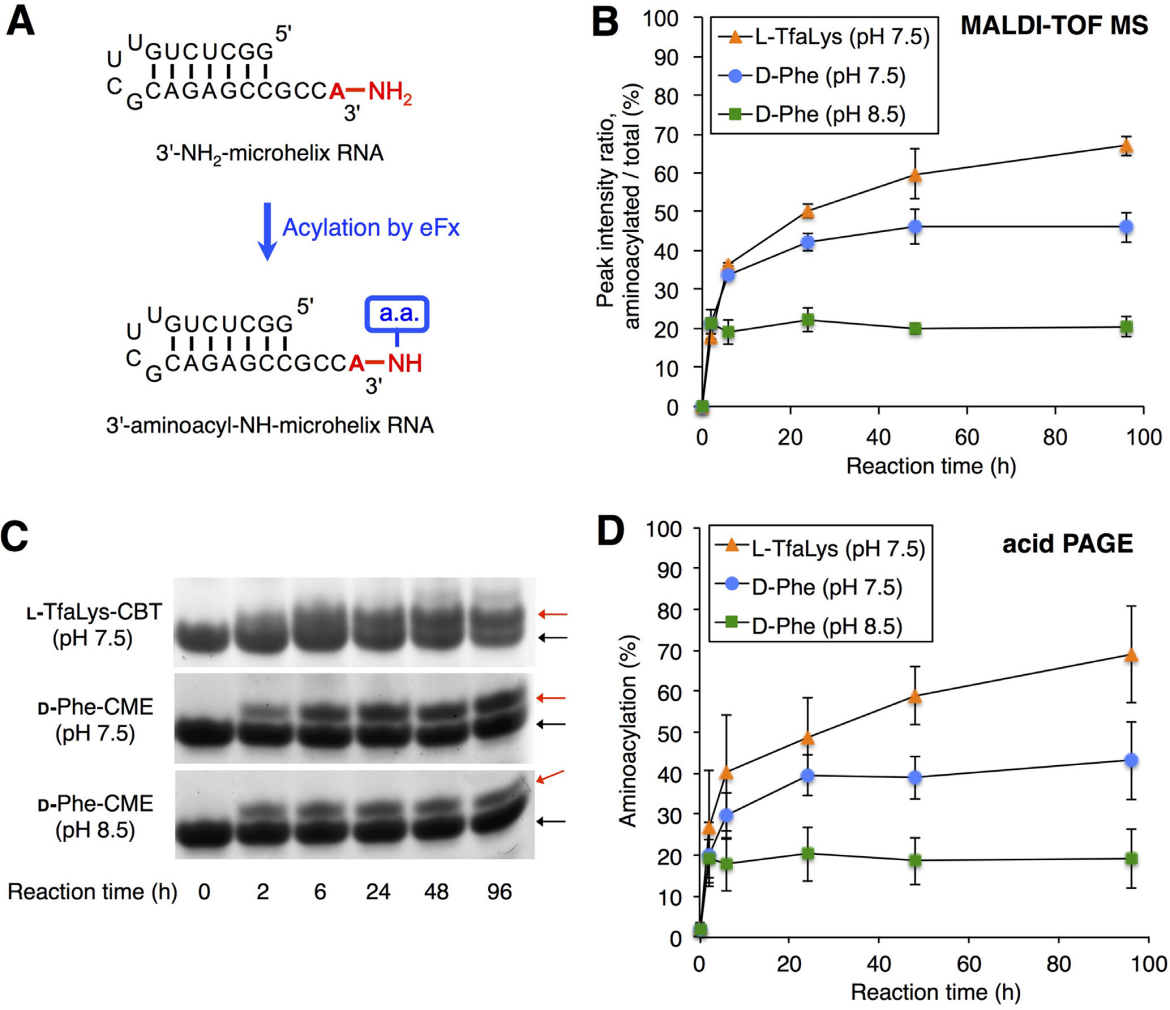
Aminoacylation of the 3′-NH_2_-microhelix. (**A**) Scheme for flexizyme-catalyzed aminoacylation of the 3′-NH_2_-microhelix. (**B**) MALDI-TOF mass spectrometric analysis of aminoacylation of 3′-NH_2_-microhelix using d-Phe-CME or l-TfaLys-CBT catalyzed by eFx. Ratios of the peak intensities of MALDI-TOF MS shown in Supplementary Figure S3 are plotted. Values are calculated by the intensity of the acylated peak divided by the sum of non-acylated and acylated peaks; *n* = 3, error bars; s.d. (**C**) Denaturing acid PAGE of the aminoacyl-microhelix charged with d-Phe or l-TfaLys. Bands were detected by ethidium bromide staining. Red arrows indicate the positions of 3′-aminoacyl-NH-microhelices. Black arrows are the positions of unreacted 3′-NH_2_-microhelices. (**D**) Graphical presentation of band intensity ratios over time, based on acid PAGE analyses as shown in (C). Values are calculated by the intensity of the acylated band divided by the sum of non-acylated and acylated band; *n* = 3, error bars; s.d.

Since the flexizymes are able to utilize not only tRNAs but also virtually any RNAs with a 3′-CCA as substrates, we next tested aminoacylation of a 3′-NH_2_-microhelix RNA consisting of a 22-nt long hairpin structure with a 3′-CCA end (Figure 4A). d-Phe-CME and l-TfaLys-CBT were used for the aminoacylation of 3′-NH_2_-microhelix, applying the same reaction conditions as for acylation of the full-length tRNA. For d-Phe-CME, both pH 7.5 and 8.5 were tested. Then, the resulting aminoacyl-NH-microhelices were analyzed by MALDI-TOF MS without RNase T1 treatment. The ratios of the peak intensities, ‘acylated’ divided by ‘non-acylated + acylated’, are plotted in Figure 4B. In all cases, no decrease of the yield was observed at longer reaction times, indicating the stability of the 3′-aminoacyl-NH-microhelix. The ratios at the 96-h reaction are 46, 21 and 67% for d-Phe (pH 7.5), d-Phe (pH 8.5) and l-TfaLys, respectively, which are comparable to the values obtained by the analysis of 3′-NH_2_-tRNA acylation (Figure 3A–C, 34, 20 and 88%, respectively). Note that both eFx and dFx recognize only the 3′-CCA region, whereas the remaining part of the tRNA body is not required. Thus, the reactivity of the microhelix RNA was assumed to be identical to that of tRNA, which explains the similar aminoacylation levels of 3′-NH_2_-microhelix and 3′-NH_2_-tRNA.

Although MALDI-TOF MS is generally not suitable for absolute quantification of compounds, the relative peak intensity of two compounds, ‘acylated’ and ‘non-acylated’ fragments, in the same spectrum reflect the actual ratio of amounts included in the reaction mixture (40). Nevertheless, in order to further verify the accuracy of the values obtained by the MALDI-TOF MS analysis, we conducted estimation of aminoacylation levels by a different method. The aminoacyl-NH-microhelices prepared by the same method as above were subjected to 20% acid PAGE. Then, the intensities of acylated and non-acylated 3′-NH_2_-microhelix were estimated by ethidium bromide staining (Figure 4C), and the ratios, ‘acylated’ divided by ‘non-acylated + acylated’, were plotted in Figure 4D. The ratios at the 96-h reaction are 43, 19 and 69% for d-Phe (pH 7.5), d-Phe (pH 8.5) and l-TfaLys, respectively, which are very close to the values obtained by MALDI-TOF MS. Therefore, we concluded that the measurement by MALDI-TOF MS should be reliable enough for estimating the yields of 3′-aminoacyl-NH-tRNAs.

### Stability assay using d-phenylalanyl-NH-tRNAs

**Figure 5.**
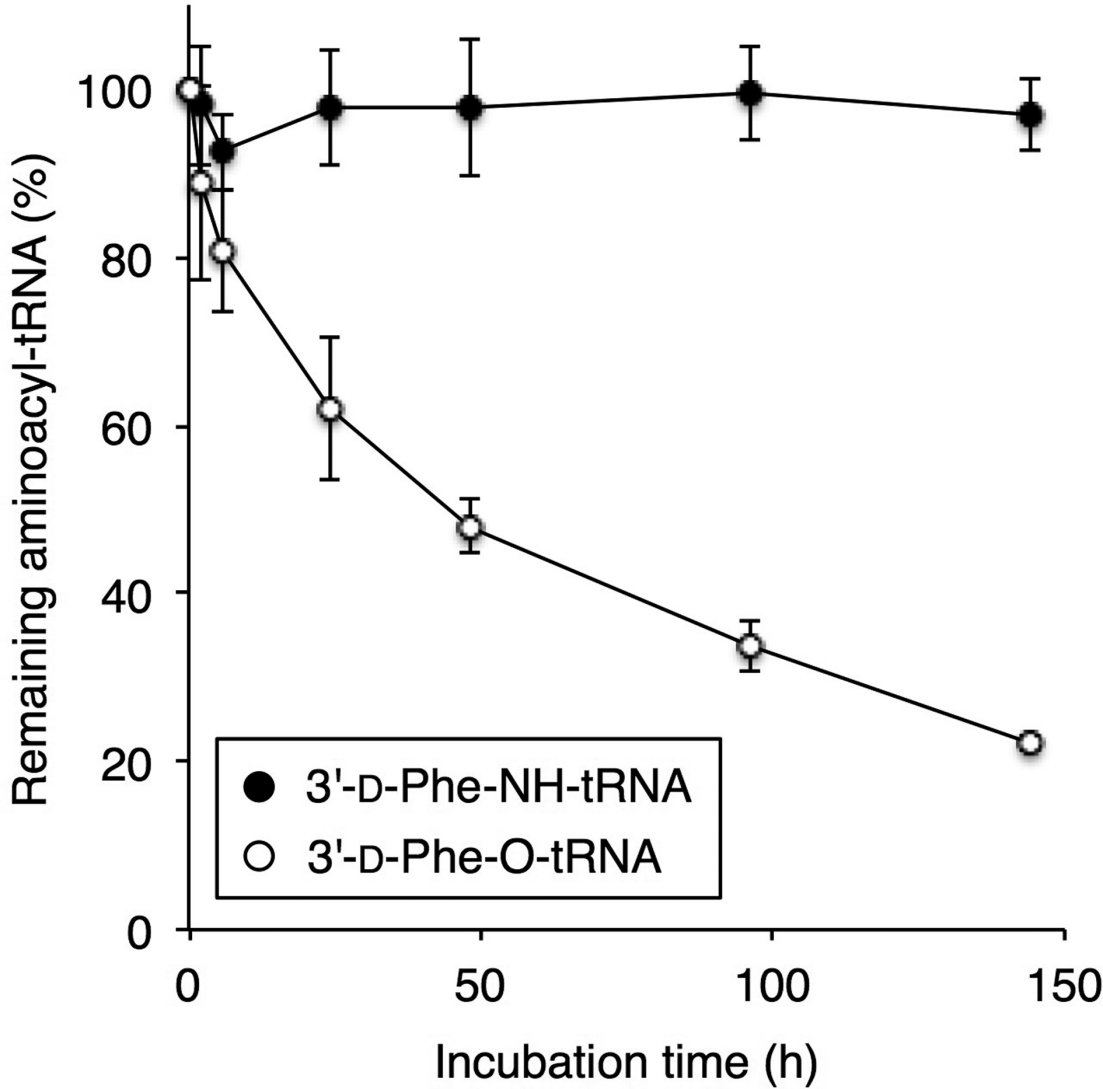
Stability assay of d-Phe-NH-tRNA and d-Phe-O-tRNA. d-Phe was charged onto 3′-NH_2_- or 3′-OH-tRNA^Tyr^ in a 96-h or a 2-h reaction, respectively. Then, d-Phe-CME was removed by ethanol precipitation. The aminoacyl-tRNAs were resuspended in 50 mM Tris–HCl (pH 7.5), and incubated at 4°C for 0, 2, 6, 24, 48, 96 and 144 h. The aminoacylation efficiencies of the RNase T1-treated fragments were estimated by MALDI-TOF MS. Values are calculated by the intensity of the acylated peak divided by the sum of non-acylated and acylated peak; *n* = 3, error bars; s.d. The values at 0 h were set to 100%.

Next, we conducted a time-course analysis to test the stability of 3′-aminoacyl-NH-tRNA. d-Phe-NH-tRNA and d-Phe-O-tRNA were synthesized under the same reaction conditions as the experiment shown in Figure 3 with 96 and 2 h of incubation, respectively, followed by removal of d-Phe-CME by ethanol precipitation and redissolving the RNA in 50 mM Tris–HCl (pH 7.5). Then, the resulting d-Phe-NH-tRNA or d-Phe-O-tRNA were incubated at 4°C for 0, 2, 6, 24, 48, 96 and 144 h, and the aminoacylation levels of the RNase T1-treated fragments were estimated by MALDI-TOF MS (Figure 5 and Supplementary Figure S4). Note that the values at 0 h are defined as 100%. As expected, no significant change in the ratio of d-Phe-NH-tRNA to NH_2_-tRNA was observed after incubation as long as 144 h, whereas most of the d-Phe-O-tRNA underwent deacylation over the time course of 144 h (<20% left). This result shows the robust hydrolytic stability of 3′-aminoacyl-NH-tRNA.

### Electrophoresis mobility shift assay of EF-Tu bound to 3′-aminoacyl-NH-tRNAs

**Figure 6.**
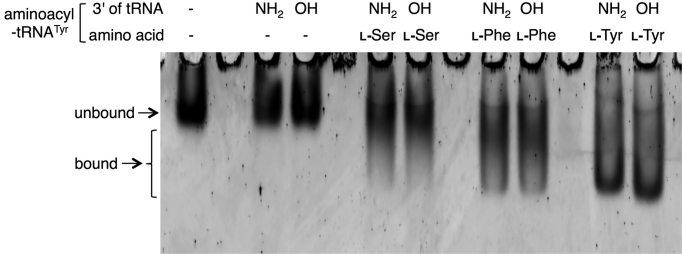
Electrophoresis mobility shift assay of EF-Tu bound to 3′-aminoacyl-NH-tRNAs. l-Ser, l-Phe and l-Tyr were charged onto 3′-NH_2_- or 3′-OH-tRNA^Tyr^ in a 96-h or a 2-h reaction, respectively. Then, 50 pmol aminoacyl-tRNAs were mixed with 50 pmol EF-Tu for the complex formation and analyzed by 8% native PAGE. Bands of the EF-Tu were detected by SYPRO Red staining. Arrows indicate the positions of unbound and bound EF-Tu. As negative controls, tRNA (-) and the use of non-acylated 3′-NH_2_- or 3′-OH-tRNA^Tyr^ were also tested for the complex formation.

Although the 3′-aminoacyl-NH-tRNA prepared by this method should not be fully functional as a component of the ribosomal translation system due to the non-canonical amide bond, it is important to confirm that this molecule can be used as a structural mimic of the canonical 3′-aminoacyl-O-tRNA. To this end, we analyzed the EF-Tu-binding ability of the 3′-aminoacyl-NH-tRNAs. l-Ser, l-Phe and l-Tyr were charged onto 3′-NH_2_- or 3′-OH-tRNA^Tyr^ by a 96-h or a 2-h flexizyme reaction, using l-Ser-DBE, l-Phe-CME and l-Tyr-CME, respectively. Then, equal amounts of aminoacyl-tRNA and EF-Tu/GTP were mixed and incubated at 37°C for 10 min to form EF-Tu/GTP/aminoacyl-tRNA ternary complex, followed by 8% native PAGE. Bands of the EF-Tu were detected by SYPRO Red staining (Figure 6). The observed mobility shifts of EF-Tu bound to l-Ser-NH-tRNA, l-Phe-NH-tRNA and l-Tyr-NH-tRNA were identical to those of EF-Tu bound to l-Ser-O-tRNA, l-Phe-O-tRNA and l-Tyr-O-tRNA, respectively, whereas the negative controls using no tRNA or non-acylated 3′-NH_2_- or 3′-OH-tRNA^Tyr^ for the complex formation exhibited no band shift. These results indicate that the 3′-aminoacyl-NH-tRNAs prepared by our method are recognized by EF-Tu.

## DISCUSSION

Here, we showed that eFx and dFx are applicable to charging various amino acids onto 3′-NH_2_-tRNA and 3′-NH_2_-microhelix RNA substrates. eFx is capable of charging CME- and CBT-activated amino acids, whereas dFx charges DBE-activated ones. CME activation is usually used for aromatic amino acids, and CBT and DBE are used for activation of non-aromatic ones, owing to the solubility of the activated amino acids in the aminoacylation reaction buffer. The results show that the yields of 3′-aminoacyl-NH-tRNA differ depending on the type of amino acid. Generally, reaction of 3′-NH_2_-tRNA is slower than that of 3′-OH-tRNA. However, since the 3′-aminoacyl-NH-tRNAs are essentially not hydrolyzed at all, the yield can be improved by extending the reaction time up to 96 h or more (Figure 2). Futhermore, 3′-aminoacyl-O-tRNAs were rapidly hydrolyzed during the reaction, and therefore the yield decreased upon increasing the reaction time from 2 to 6 h or longer.

One important advantage of using flexizymes is that not only proteinogenic but also nonproteinogenic amino acids can be used as substrates for aminoacylation. The combination of amino acids and tRNAs is by far not as limited as in approaches utilizing proteinaceous ARSs. Here, we showed that d-Phe, l-IodoPhe, l-MePhe, l-AcPhe, l-TfaLys, l-MeVal and d-Ala can be charged onto 3′-NH_2_-tRNA. Flexizymes are capable of charging even more diverse substrates, including β- and γ-amino acids, hydroxy acids, side-chain modified ones etc. onto the canonical 3′-OH-tRNAs. Although such amino acid substrates were not tested in this work, it is likely that they can also be used for charging of 3′-NH_2_-tRNAs. As the flexizymes do not recognize the body sequence of tRNA but only the conserved 3′-CCA region, not only tRNAs but also any RNAs with a 3′-CCA sequence can be used as substrates in the flexizyme reaction. Indeed, we showed that the microhelix RNA, a hairpin RNA with 3′-CCA end and the 3′-amino group, could be efficiently charged with amino acids by the flexizymes. Consequently, flexizymes provide an easy and versatile method for preparing diverse unhydrolyzable 3′-aminoacyl-NH-RNAs in parallel.

Due to the importance of aminoacyl-tRNAs in ribosomal translation, structural and functional analysis of aminoacyl-tRNAs in complex with ribosomes has been of great interest to researchers in this field for a long time. The nonproteinogenic 3′-aminoacyl-NH-tRNAs synthesized by flexizymes certainly will expand such studies and potentially reveal new insights into why ribosomes can accommodate some types of nonproteinogenic amino acids in their active site, while acting less efficiently on others. Moreover, aminoacyl-tRNAs are also utilized in other cellular reactions like transfer of amino acids to certain other molecules. For instance, bacterial aminoacyl-phosphatidylglycerol synthases (aaPGSs) and Fem family proteins are involved in transfer of amino acids from specific aminoacyl-tRNAs to phosphatidylglycerol and peptidoglycan, respectively (41,42). Therefore, structural analysis of aminoacyl-tRNAs bound to such enzymes would be of great interest. For such purposes, appropriate hydrolysis-resistant 3′-aminoacyl-NH-tRNAs should be useful for preventing undesired deacylation during sample preparation. As 3′-aminoacyl-NH-tRNAs prevent 3′-O/2′-O transesterification, it can also be used for fixing the position of amino acids at the 3′ position.

## Supplementary Material

Supplementary Data
