# Supplementary material for: Flexizyme-catalyzed synthesis of 3′-aminoacyl-NH-tRNAs
Source: Nucleic Acids Res. 2019 Mar 7;47(9):e54. doi: 10.1093/nar/gkz143 (PMC6511858; doi:10.1093/nar/gkz143)
Supplement: Supplementary Data [file gkz143_supplemental_files.zip › 190124_amide-tRNA-supplementary.pptx.pdf]

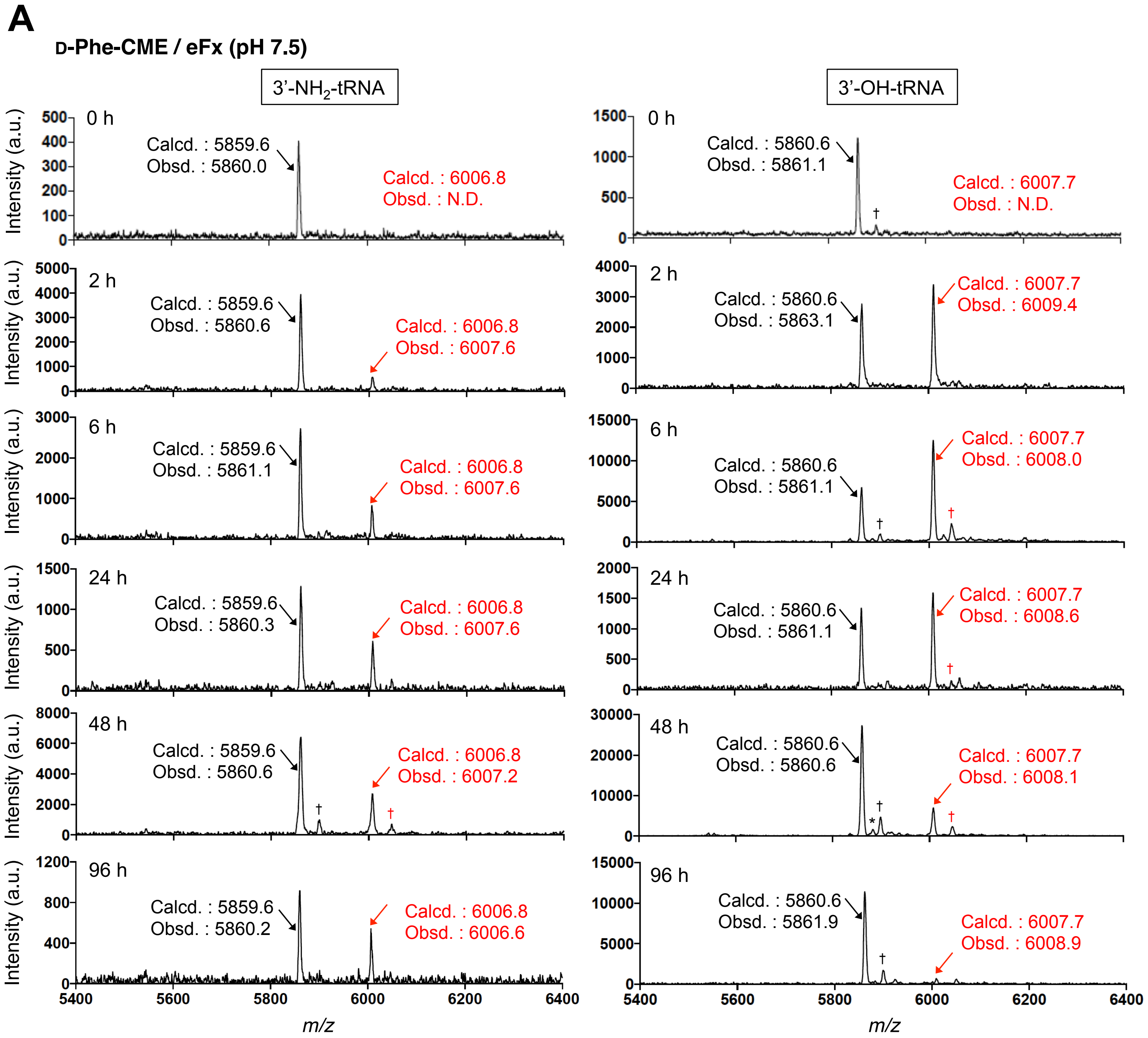

Supplementary Figure S1. MALDI-TOF mass spectra of RNase T1-digested aminoacyl-tRNA fragments. D-Phe-CME and L-TfaLys-CBT were charged by eFx, and L-Ser-DBE by dFx on to *E. coli* $tRNA^{Tyr}$ bearing a 3'-amino group (3'-NH_2-tRNA) or a 3'-hydroxy group (3'-OH-tRNA). Reactions were performed at pH 7.5 and also at pH 8.5 for D-Phe-CME. **A)** D-Phe-CME (pH 7.5). **B)** D-Phe-CME (pH 8.5). **C)** L-TfaLys-CBT (pH 7.5). **D)** L-Ser-DBE (pH 7.5). Then, the aminoacyl-tRNA was digested with RNase T1 and analyzed by MALDI-TOF MS. Red arrows indicate the peaks of the fragments derived from aminoacyl-tRNA, and black arrows are those of non-acylated ones. Calculated and observed *m/z* values of the $[M+H]^+$ ions are shown. * and † indicate the $[M+Na]^+$ and $[M+K]^+$ ions, respectively. See also figure 3 for quantification of the peak intensities.

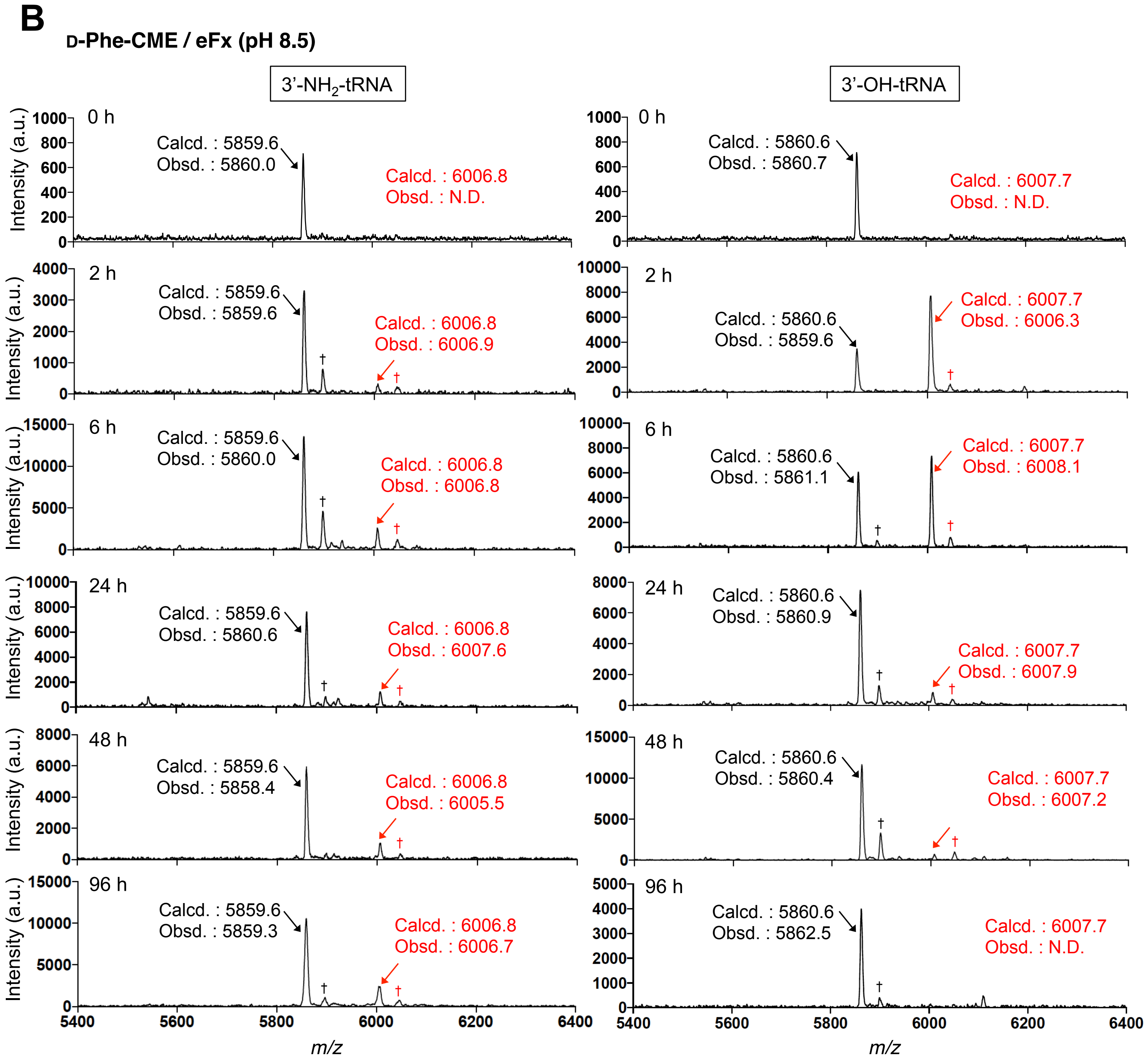

Supplementary Figure S1, continued. MALDI-TOF mass spectra of RNase T1-digested aminoacyl-tRNA fragments. D-Phe-CME and L-TfaLys-CBT were charged by eFx, and L-Ser-DBE by dFx on to *E. coli* tRNA^{Tyr} bearing a 3'-amino group (3'-NH_2-tRNA) or a 3'-hydroxy group (3'-OH-tRNA). Reactions were performed at pH 7.5 and also at pH 8.5 for D-Phe-CME. **A)** D-Phe-CME (pH 7.5). **B)** D-Phe-CME (pH 8.5). **C)** L-TfaLys-CBT (pH 7.5). **D)** L-Ser-DBE (pH 7.5). Then, the aminoacyl-tRNA was digested with RNase T1 and analyzed by MALDI-TOF MS. Red arrows indicate the peaks of the fragments derived from aminoacyl-tRNA, and black arrows are those of non-acylated ones. Calculated and observed *m/z* values of the $[M+H]^+$ ions are shown. * and † indicate the $[M+Na]^+$ and $[M+K]^+$ ions, respectively. See also figure 3 for quantification of the peak intensities.

C

L-TfaLys-CBT / eFx (pH 7.5)

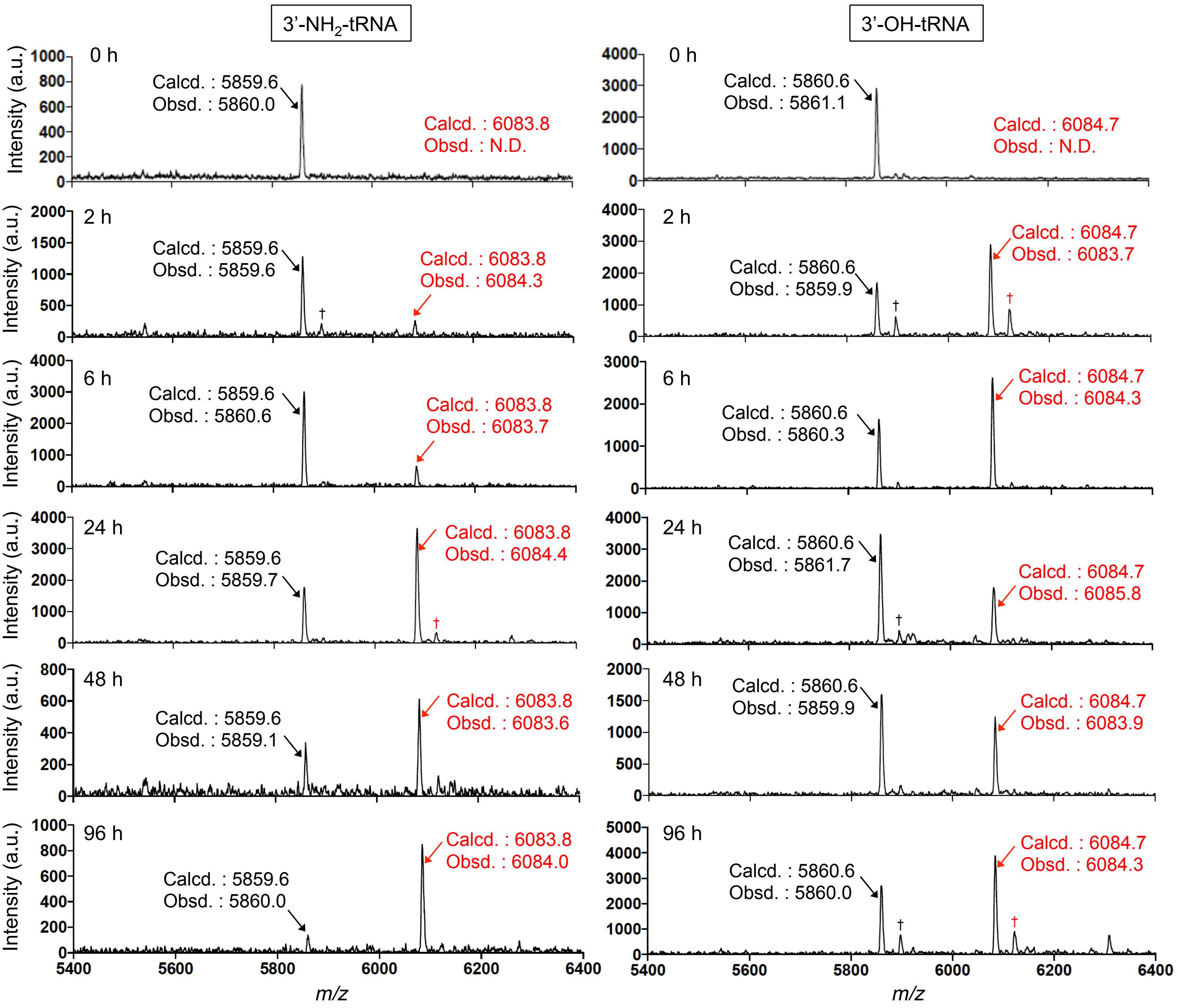

Supplementary Figure S1, continued. MALDI-TOF mass spectra of RNase T1-digested aminoacyl-tRNA fragments. D-Phe-CME and L-TfaLys-CBT were charged by eFx, and L-Ser-DBE by dFx on to *E. coli* tRNA^{Tyr} bearing a 3'-amino group (3'-NH$_2$-tRNA) or a 3'-hydroxy group (3'-OH-tRNA). Reactions were performed at pH 7.5 and also at pH 8.5 for D-Phe-CME. **A)** D-Phe-CME (pH 7.5). **B)** D-Phe-CME (pH 8.5). **C)** L-TfaLys-CBT (pH 7.5). **D)** L-Ser-DBE (pH 7.5). Then, the aminoacyl-tRNA was digested with RNase T1 and analyzed by MALDI-TOF MS. Red arrows indicate the peaks of the fragments derived from aminoacyl-tRNA, and black arrows are those of non-acylated ones. Calculated and observed *m/z* values of the $[M+H]^+$ ions are shown. * and † indicate the $[M+Na]^+$ and $[M+K]^+$ ions, respectively. See also figure 3 for quantification of the peak intensities.

D **L-Ser-DBE / dFx (pH 7.5)**

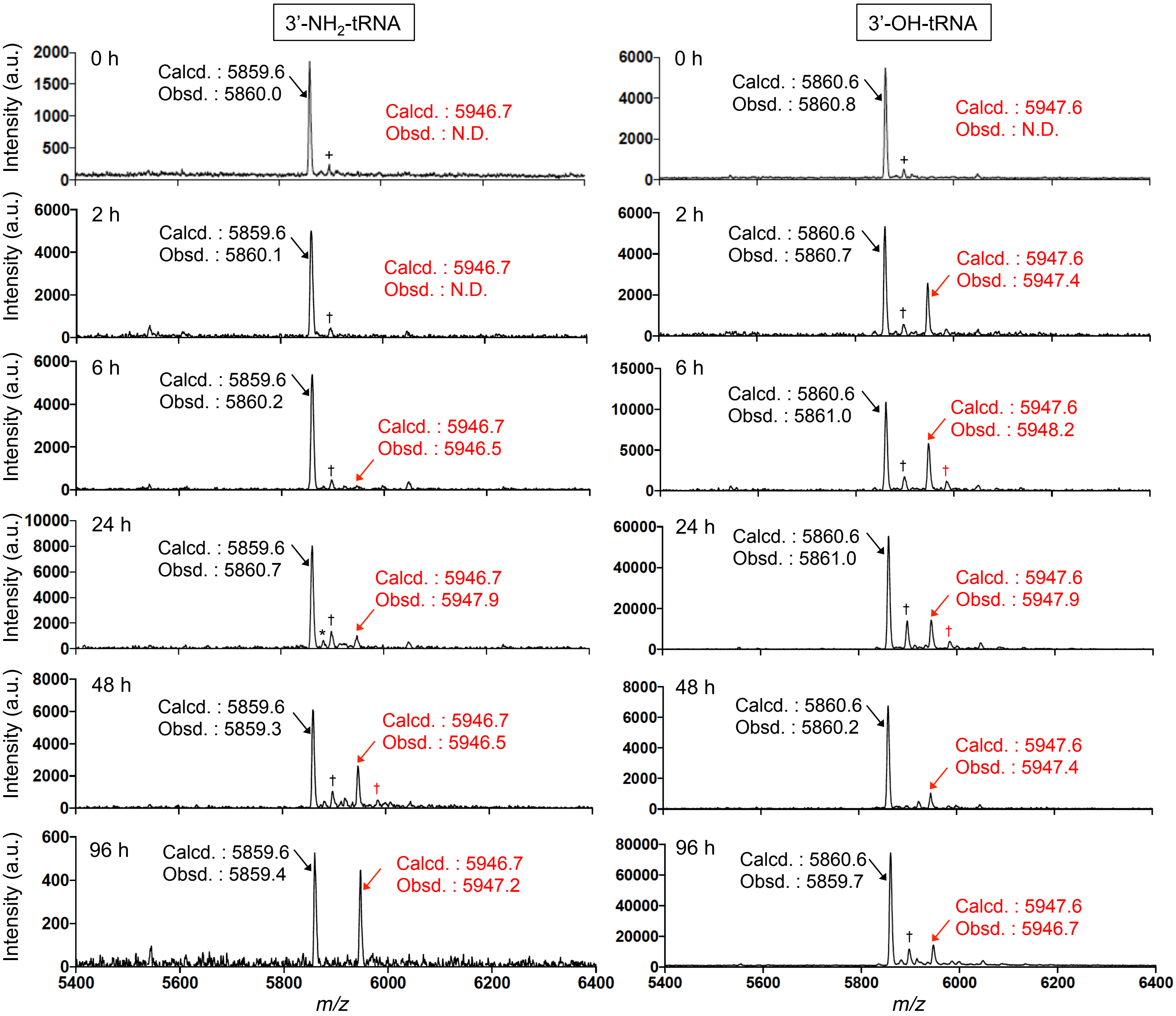

Supplementary Figure S1, continued. MALDI-TOF mass spectra of RNase T1-digested aminoacyl-tRNA fragments. D-Phe-CME and L-TfaLys-CBT were charged by eFx, and L-Ser-DBE by dFx on to *E. coli* $tRNA^{Tyr}$ bearing a 3'-amino group (3'-NH_2-tRNA) or a 3'-hydroxy group (3'-OH-tRNA). Reactions were performed at pH 7.5 and also at pH 8.5 for D-Phe-CME. **A)** D-Phe-CME (pH 7.5). **B)** D-Phe-CME (pH 8.5). **C)** L-TfaLys-CBT (pH 7.5). **D)** L-Ser-DBE (pH 7.5). Then, the aminoacyl-tRNA was digested with RNase T1 and analyzed by MALDI-TOF MS. Red arrows indicate the peaks of the fragments derived from aminoacyl-tRNA, and black arrows are those of non-acylated ones. Calculated and observed *m/z* values of the $[M+H]^+$ ions are shown. * and † indicate the $[M+Na]^+$ and $[M+K]^+$ ions, respectively. See also figure 3 for quantification of the peak intensities.

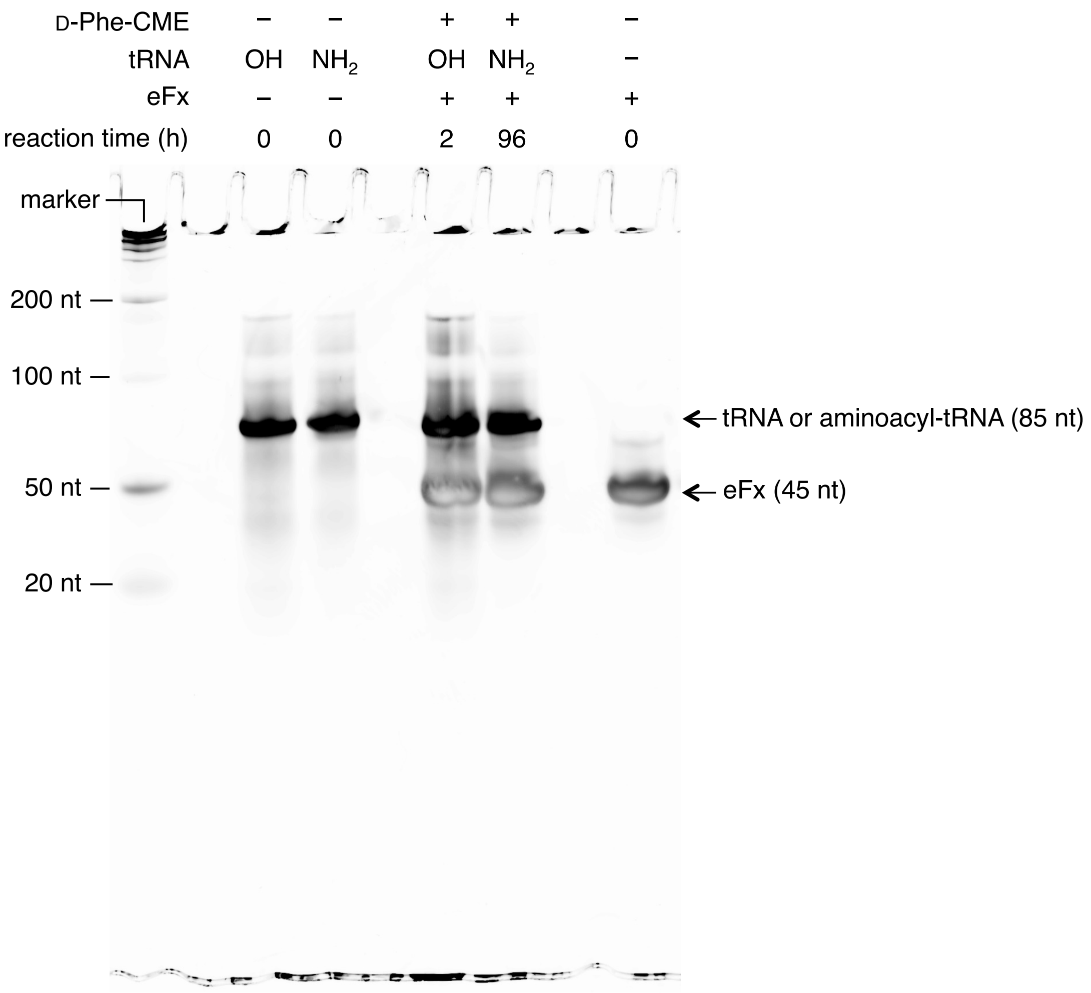

Supplementary Figure S2. Analysis of the hydrolytic stability of tRNA body under the flexizyme reaction conditions. D-Phe-O-tRNA and D-Phe-NH-tRNA were prepared by a 2-h or a 96-h flexizyme reaction, respectively, at pH 7.5 using eFx, and analyzed by 10% acid PAGE containing 6 M urea at 120 V for 45 min. Unreacted tRNAs and eFx were also applied to the gel as controls. Note that the bands of aminoacyl-tRNA and non-acylated tRNA cannot be separated under these analytical conditions.

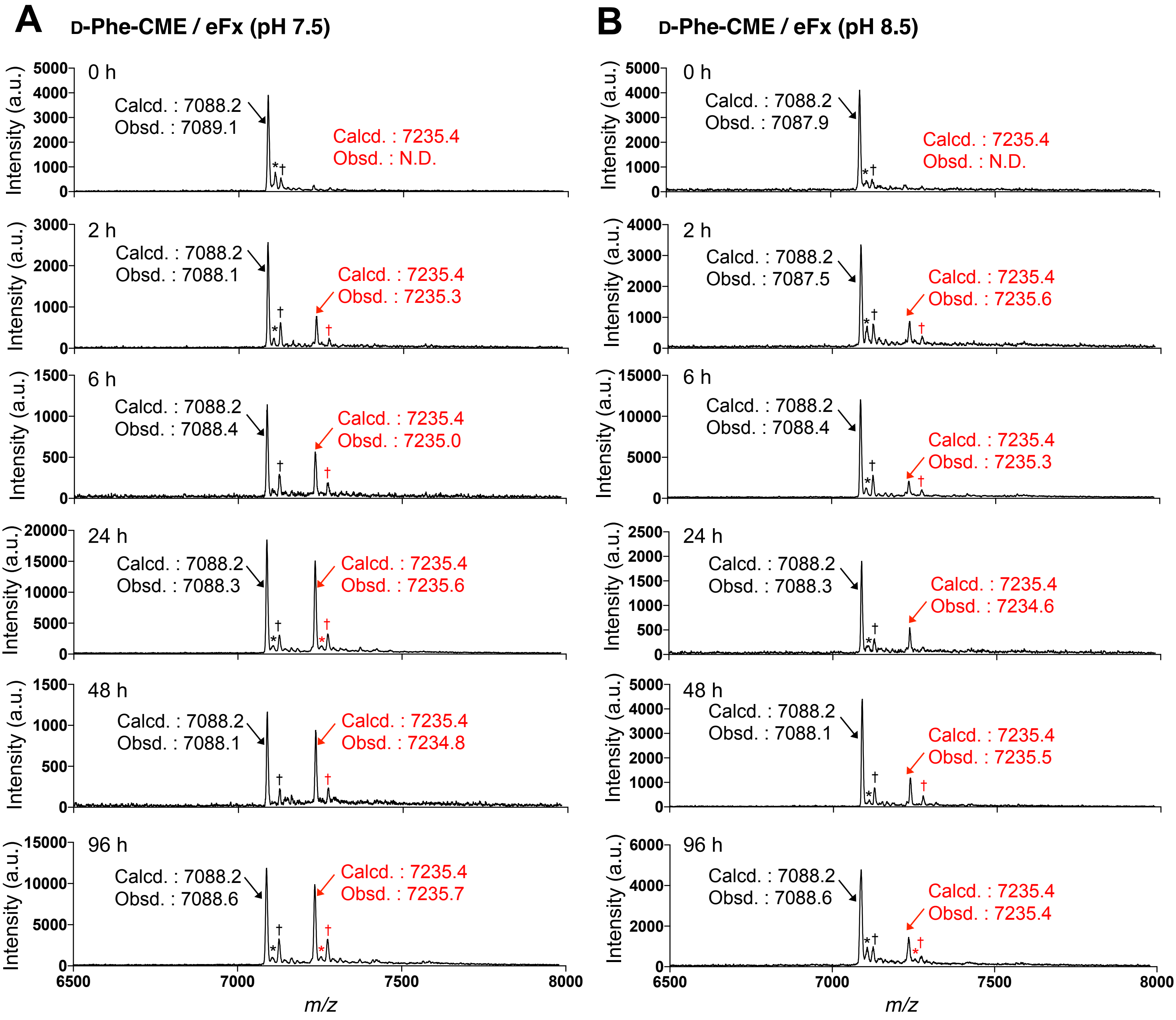

Supplementary Figure S3. MALDI-TOF mass spectra of aminoacyl-NH-microhelices. D-Phe-CME and L-TfaLys-CBT were charged by eFx onto the microhelix RNA bearing a 3'-amino group. Reactions were performed at pH 7.5 and also at pH 8.5 for D-Phe-CME. **A)** D-Phe-CME (pH 7.5). **B)** D-Phe-CME (pH 8.5). **C)** L-TfaLys-CBT (pH 7.5). Red arrows indicate the peaks of the aminoacyl-NH-microhelices, and black arrows are those of non-acylated ones. Calculated and observed *m/z* values of the $[M+H]^+$ ions are shown. * and † indicate the $[M+Na]^+$ and $[M+K]^+$ ions, respectively. See also figure 4B for quantification of the peak intensities.

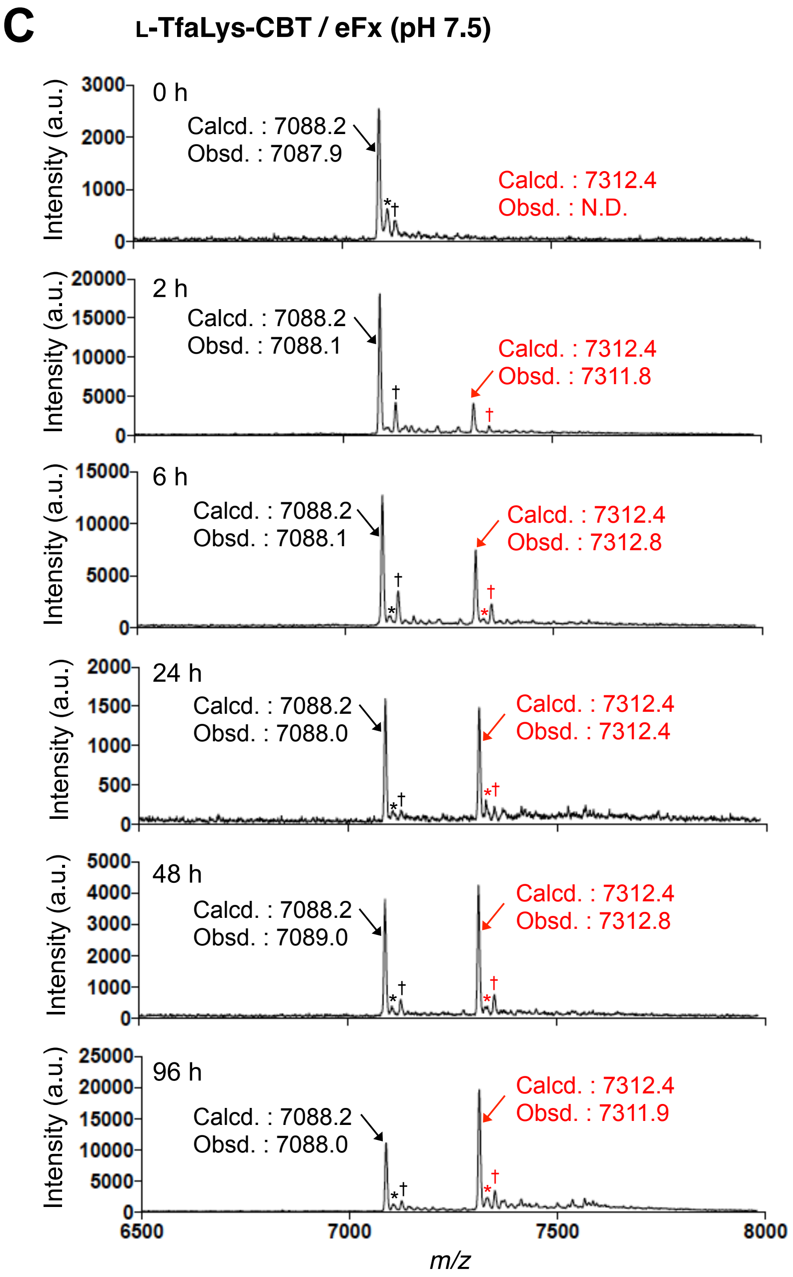

Supplementary Figure S3, continued. MALDI-TOF mass spectra of aminoacyl-NH-microhelices. D-Phe-CME and L-TfaLys-CBT were charged by eFx onto the microhelix RNA bearing a 3'-amino group. Reactions were performed at pH 7.5 and also at pH 8.5 for D-Phe-CME. **A)** D-Phe-CME (pH 7.5). **B)** D-Phe-CME (pH 8.5). **C)** L-TfaLys-CBT (pH 7.5). Red arrows indicate the peaks of the aminoacyl-NH-microhelices, and black arrows are those of non-acylated ones. Calculated and observed *m/z* values of the $[M+H]^+$ ions are shown. * and † indicate the $[M+Na]^+$ and $[M+K]^+$ ions, respectively. See also figure 4B for quantification of the peak intensities.

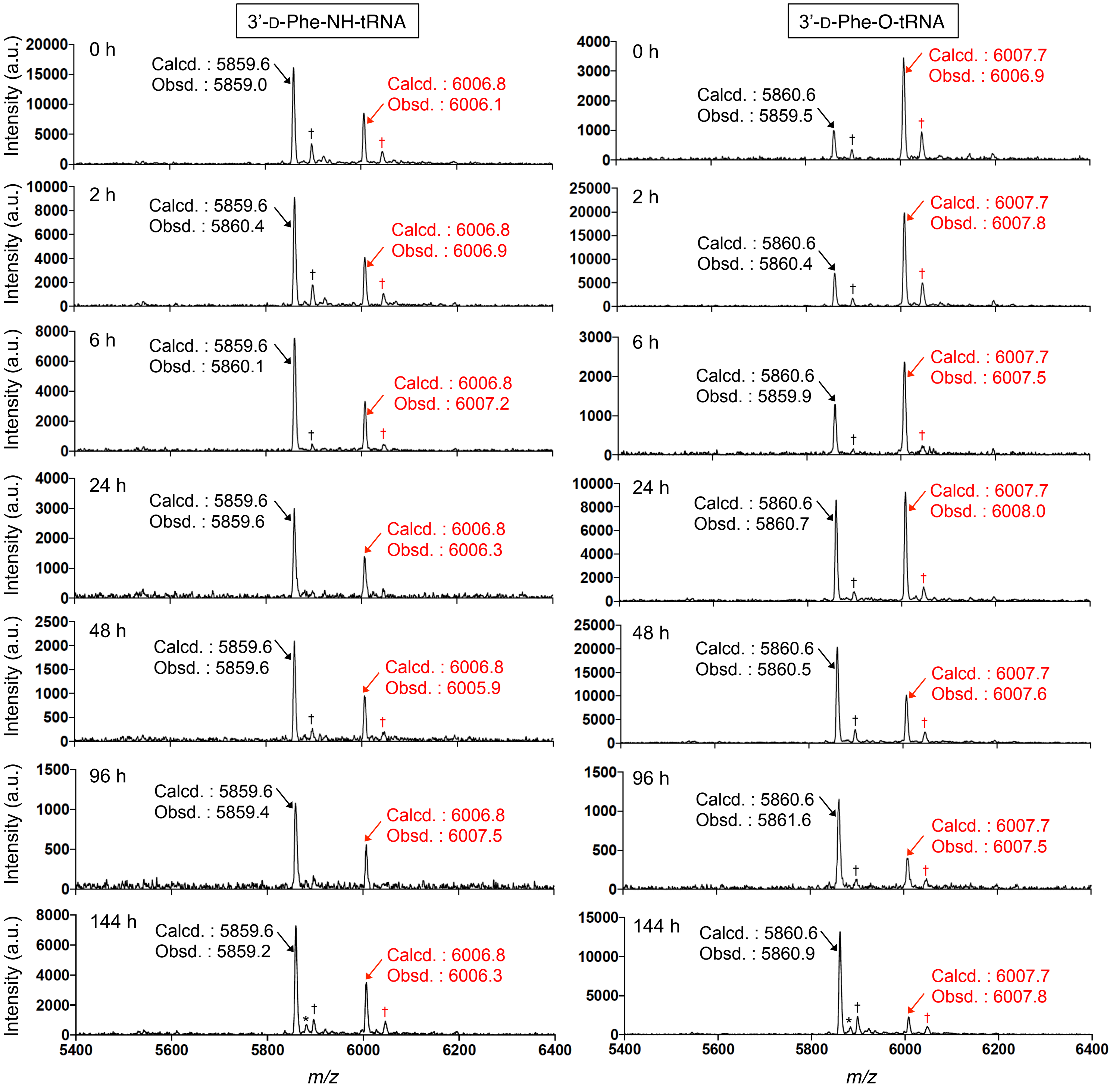

Supplementary Figure S4. Stability assay of aminoacyl-tRNAs. D-Phe was charged on to *E. coli* $tRNA^{Tyr}$ bearing a 3'-amino group (3'-D-Phe-NH-tRNA) or a 3'-hydroxy group (3'-D-Phe-O-tRNA) with 96 h and 2 h reaction, respectively. Then, D-Phe-CME was removed by ethanol precipitation. The aminoacyl-tRNAs were resuspended in 50 mM Tris-HCl (pH 7.5), and incubated at 4˚C for 0, 2, 6, 24, 48, 96, and 144 h. Then, the aminoacyl-tRNA was digested with RNase T1 and analyzed by MALDI-TOF MS. Red arrows indicate the peaks of the fragments derived from aminoacyl-tRNA, and black arrows are those of non-acylated ones. Calculated and observed *m/z* values of the $[M+H]^+$ ions are shown. * and † indicate the $[M+Na]^+$ and $[M+K]^+$ ions, respectively. See also figure 5 for quantification of the peak intensities.
